# Structural and mechanical properties of a giomer-based bulk fill restorative in different curing conditions

**DOI:** 10.1590/1678-7757-2016-0662

**Published:** 2018-01-16

**Authors:** Mustafa Sarp Kaya, Meltem Bakkal, Ali Durmus, Zehra Durmus

**Affiliations:** 1 Bezmialem Vakif University, Faculty of Dentistry, Department of Pediatric Dentistry, Istanbul, Turkey; 2 Istanbul University, Faculty of Engineering, Department of Chemical Engineering, Istanbul, Turkey; 3 Bezmialem Vakıf University, Faculty of Pharmacy, Department of Pharmaceutical Biotechnology, Istanbul, Turkey

**Keywords:** Dental resins, Fourier-transform infrared spectroscopy, Hardness tests, Polymerization

## Abstract

**Objective:**

The main goal of this study was to compare the polymerization degree of bulk-fill giomer resin cured with three different light-curing units (LCUs): a polywave third-generation (Valo); a monowave (DemiUltra: DU); and a second-generation LED (Optima 10: Opt) LCUs by using structural and mechanical properties.

**Material and methods:**

Giomer samples of 2 and 4 mm cured with three LCUs were employed *in vitro* analysis. The degree of curing (DC%) was determined with Fourier-Transform Infrared Spectroscopy (FTIR). Microstructural features were observed with scanning electron microscopy (SEM). Flexural strength (FS), compression strength (CS), elastic modulus and fracturing strain were determined for mechanical properties. Surface microhardness (SMH) values were also measured. Oneway ANOVA, two-way analysis of variance and Tukey multiple comparison tests were used for statistically analyzing the FS and SMH.

**Results:**

DC% values were 58.2, 47.6, and 39.7 for the 2 mm samples cured with DU, Opt., and Valo LCUs, respectively. DC% values of the 4 mm samples were 50.4, 44.6, and 38.2 for DU, Opt, and Valo, respectively. SMH values were Valo, Opt<DU at top of the samples; Valo<DU, Opt at 2 mm, and DU, Valo<Opt at 4 mm depth. Giomer samples cured with Opt and DU exhibited higher FS values than Valo. CS values were similar but compressive modulus and fracturing strain (%) varied depending on the curing protocol.

**Conclusions:**

Based on the results, it can be concluded that curing device and protocol strongly affect crosslinking reactions and thus DC%, SMH, compressive modulus and strain at break values. Consequently, it can be deduced that curing protocol is possibly the most important parameter for microstructure formation of highly-filled composite restoratives because it may bring some structural defects and physical frailties on restorations due to lower degree of polymerization.

## Introduction

Bulk-fill resins have been developed to speed up the emplacement of restorative material. These new restorative composites can be used to fill cavities by single or multiple increments^6^. Bulk-fill restoratives generally include small-sized or lower amount of fillers to decrease light scattering. This structural feature allows the material to be applied up to 4 mm increments at a time. Innovation in bulk fills introduced various new photoinitiators and fillers^16^.

Giomer is an alternative novel hybrid dental restorative material containing pre-reacted glass- ionomer filler particles in a resin matrix that provides some advantages for fluoride releasing and recharging, in addition to enhanced mechanical, esthetical and handling properties^5^. Recently a bulk-fill giomer in low viscosity and high viscosity forms was introduced, claiming a combination of anticarious properties, esthetic, durability and fast-treatment comfort^13^.

Light-cured resin materials allow controlling curing time but also require incremental polymerization, which has been suggested to be restricted to 2 mm until recently for the majority of composites with most of the light-curing unit (LCU) in the market^7^ ^,^ ^8^. In addition to the compositional improvements in resin phase and polymerization issues, some LCU manufacturers have claimed to decrease curing time and thus treatment period by increasing the irradiance outputs (mW/cm^2^) of their equipment, following the assumption that radiant exposure has a simple reciprocal relationship: if the irradiance is increased, the light-curing time can be decreased^18^ ^,^ ^26^. Most recent advancements in curing technology have appeared in light-emitting diode (LED) LCUs. These devices have become popular due to their several operational advantages such as shorter exposure times, longer service time, lower weight and thermal effects compared to halogen lights and ultraviolet (UV) predecessors^11^ ^,^ ^19^ ^,^ ^24^. First- and second-generation LED-LCUs were able to polymerize 2 mm thick resin samples in 20-40 s and emitted a narrow monowave light spectrum (450-470 nm), which corresponds to the spectral peak absorbance of camphorquinone. However, some resin manufacturers have started to use alternative photoinitiators, which necessitated suitable LCU. Recently, third-generation LED-LCUs were developed, which can emit multi-wave light to activate multi-component photo-initiator systems with high irradiance outputs and provide sufficient polymerization with shorter curing^11^ ^,^ ^19^ ^,^ ^24^.

Polymerization of dental restoratives can be determined indirectly by scraping methods, depth of cure and surface microhardness (SMH) tests or directly with Fourier-Transform Infrared Spectroscopy (FTIR). FTIR is a spectroscopic technique used to analyze the chemical bonds of polymers by comparing the peaks of C=C bands. Although FTIR has been reported to be a superior measure for quantifying polymerization, it is a complex, high-cost and time-consuming method^23^. SMH is a very common and simpler method, which uses the measurements of its specific indenter to test polymerization and has been reported as a good indicator of polymerization^7^ ^,^ ^8^ ^,^ ^11^.

Insufficiently polymerized resins have been reported to perform decreased mechanical properties (flexural and fracture strengths), wear resistance, bond strength, low color stability and predispose pulp irritation with unpolymerized monomers^3^ ^,^ ^22^ ^,^ ^27^. Flexural strength (FS) (ISO 4049:2009) is a frequently used standard mechanical test indicative of clinical performance^1^ ^,^ ^13^ ^,^ ^17^.

Considering the novelty of bulk-fill giomer restorative and third-generation LCUs, quantitative relationships between microstructure formation and resulting physical properties of restoratives and curing protocols should be studied in detail.

The aim of this study was to compare the polymerization degree of bulk-fill giomer resin cured with three different light-curing units (LCUs): a polywave third-generation (Valo), a monowave (DemiUltra: DU), and a second-generation LED (Optima 10: Opt) LCUs by using structural and mechanical properties. The null hypothesis of "polymerization do not differ depending on the curing protocols" was tested.

## Material and methods

### Material

**Figure 1:**
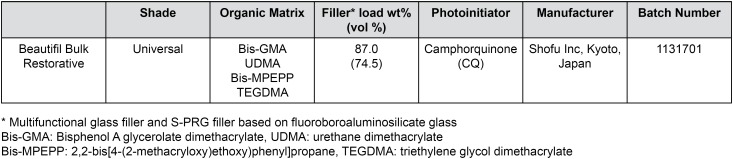
Properties of the bulk-fill giomer resin investigated

A commercial resin, Giomer (Beautifil-Bulk Restorative, Shofu Inc, Kyoto, Japan) was used in this study. According to the commercial brochure declared by the manufacturer, Giomer is a high filled, low-shrinking composite for posterior restorations including occlusal surfaces, showing excellent condensability, sculptability and shade stability. Some of the commercial information and physical properties are listed in Figure 1.

### Sample preparation

Two ring-shaped Teflon molds, with a diameter of 5 mm and thickness of 2 and 4 mm, were used to prepare six groups of samples. Giomer samples cured with DemiUltra, Optima 10, and Valo are henceforth denoted as G-DU, G-Opt and G-Valo, respectively.

**Figure 2:**
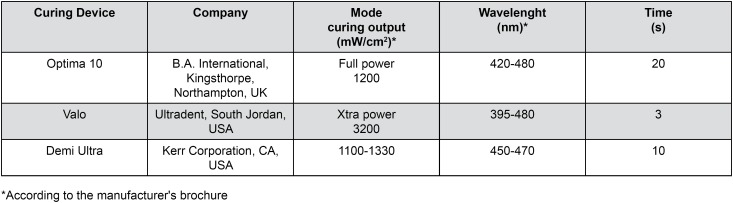
Curing devices and polymerization protocols used in this study

Giomer was placed into molds and covered with a Mylar Strip and then light-cured with different LCU units. Three protocols were applied, as showed in Figure 2.

A second-generation monowave LED unit Optima 10 (420-480 nm, 1200 mW/cm^2^, B.A. International, Kingsthorpe, Northampton, UK) was used for 20 s in full power mode to simulate conventional curing conditions;

A third-generation multiwave Valo LED (395-480 nm, 3200 mW/cm^2^, Ultradent, South Jordan, UT, USA) was used for 6 s in Xtra power mode to simulate short curing time claimed by manufacturer;

DemiUltra (450-470 nm, 1100-1300 mW/cm^2^, Kerr Corporation, Orange, CA, USA) was used for 10 s to simulate short curing time with a recent LED- LCU according to the manufacturer's suggestion for restoration.

Before the study, we checked the light intensity of all LCUs using a Demetron radiometer (Kerr Corporation, Orange, CA, USA).

In addition, we prepared the samples used for the flexural strength tests using rectangular molds (2×2×25 mm) compressed between two glass plates. The entire length of each specimen was irradiated by modifying the ISO 4049:2009 protocol to ensure equal curing throughout the specimen. The light tip was moved to half the diameter overlapping the previously irradiated section, along the specimen directly contacting the covering acetate strip.

### Microstructural and morphological analysis

Variations in the microstructural features of samples polymerized by employing different curing protocols were characterized by the FTIR method. FTIR spectra of samples were recorded in transmission mode with a Bruker Alpha infrared spectrometer by using an attenuated total reflectance (ATR) device and germanium crystal, within a wavenumber range of 400-4000 cm^−1^ with a resolution of 2 cm^−1^ from 32 scans. The spectra were analyzed by using the "OPUS" and Origin v8.5 software to calculate degree of conversion (DC%) values and quantify crosslinking reactions depending on the curing conditions.

The DC% value was calculated by considering relative change in characteristic peak intensities with the following equation^25^:

$$\mathrm{DC}(\%)=\left[1-\frac{{\left(A_{1636}-A_{1604}\right)}_{ac}}{{\left(A_{1636}-A_{1604}\right)}_{bc}}\right]X100$$

where *A* is the intensity of a particular absorption peak, *ac* and *bc* represent conditions of "after curing" and "before curing", respectively.

The morphological properties of a representative sample (G-DU) were investigated in a field emission scanning electron (FE-SEM, FEI Quanta FEG 450) microscope. In the SEM analysis, fractured surfaces of the sample after the compression test were directly imaged in the instrument after a proper sample preparation route sputter-coated with gold.

### Surface hardness measurement

After polymerization, top surfaces of samples were polished by using a 400, 800, 1000, 1500, 2000, 2500 grit silicon carbide (SiC) paper and were immediately tested. Five specimens were used in each LCU group and two material thicknesses (2 mm, 4 mm) for surface hardness measurements. Vickers hardness value was measured with a microhardness tester (HMV M-1, Shimadzu Corp, Kyoto, Japan). Samples were applied a constant load of 100 g for 10 s (Vickers pyramid: diamond right pyramid with a square base and an angle of a = 136° between the opposite faces at the vertex). Measurements were performed on the top and bottom surfaces of the samples (0: top, 2 or 4 mm: bottom depth). Five indentations were performed onto each sample's surface, one in the center and one in every quadrant (>100 μm from each other). Results were independently averaged and reported as SMH. Besides comparing SMH values among the groups, bottom/top ratios of each group ≥80% criteria was also used to assess microhardness as proposed in the literature^2^ ^,^ ^7^ ^,^ ^8^.

### Mechanical properties

**Figure 3:**
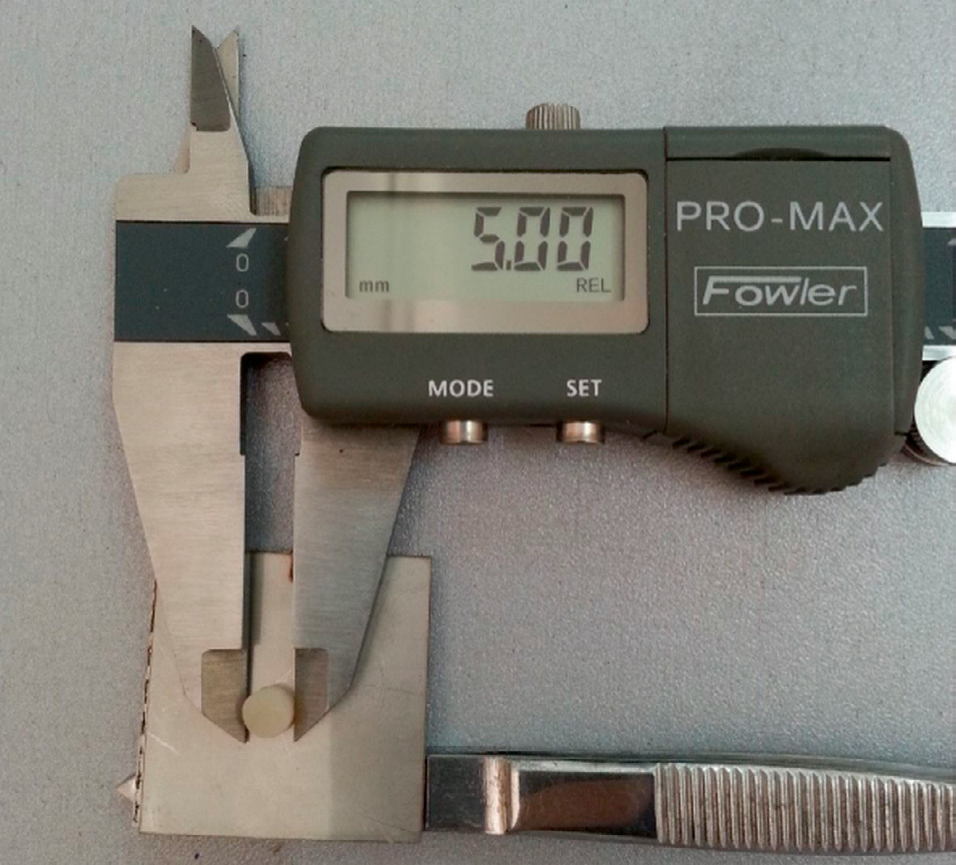
Compression test sample

Mechanical properties of samples were tested in a universal tension-compression test machine (BWB- 20, KokBir, Istanbul, Turkey) in compression mode by using the cylindrical test specimens of 5 mm diameter and 2 mm thickness. In the compression tests, cross-head or compression speed was applied as 0.1 mm/minute. Representative photographs of a molded sample used in compression tests are shown in Figure 3. Five specimens were tested in compression tests and the average values and standard deviations were reported.

Flexural strength values of samples were determined by three-point bending tests performed in a universal testing machine (Shimadzu AGS-X, Japan). Measurements were carried out by applying a constant-speed force at the point of 10 mm distance between supports with the crosshead speed of 0.5 mm/min, according to the ISO/DIN 4049:2009 standard^17^. Ten specimens cured with each LCU were tested.

### Statistical analysis

Statistical analyses were performed by using SPSS 20.0 for Windows. The normality of the distributions was confirmed by skewness, kurtosis and the Kolmogorov-Smirnov test. One-way ANOVA, two-way analysis of variance and Tukey multiple comparisons were used for comparing FS and SMH values of samples. All results were considered significant at p<0.05.

## Results

### Microstructure and morphology

**Figure 4:**
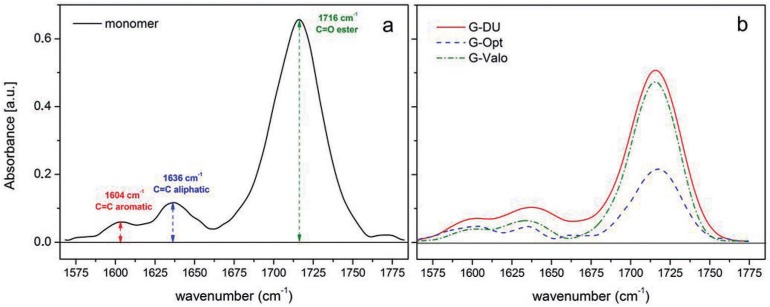
Fourier-transform infrared spectroscopy (FTIR) spectra of uncured resin (a) and cured giomer (b) by using different light sources at 2 mm depth within a narrow range of wavenumber

The FT-IR spectra of uncured resin and cured composites by using different LCUs within a narrow range of wavenumber (1570-1780 cm^−1^) are shown in Figures 4a and 4b. To quantify DC% we used the intensity of the characteristic absorption peak of the unsaturated aliphatic C=C double bond originated from the methacrylate group at 1636 cm^−1^ and that of aromatic C=C double bond at 1604 cm^−1^. DC% values of samples are listed in Table 1.

**Table 1:** DC% of giomer samples cured with different light-curing units (LCUs)

Depth	Sample	DC (%)
2 mm	G-DU	58.2
	G-Opt	47.6
	G-Valo	39.7

4 mm	G-DU	50.4
	G-Opt	44.6
	G-Valo	38.2

**Figure 5:**
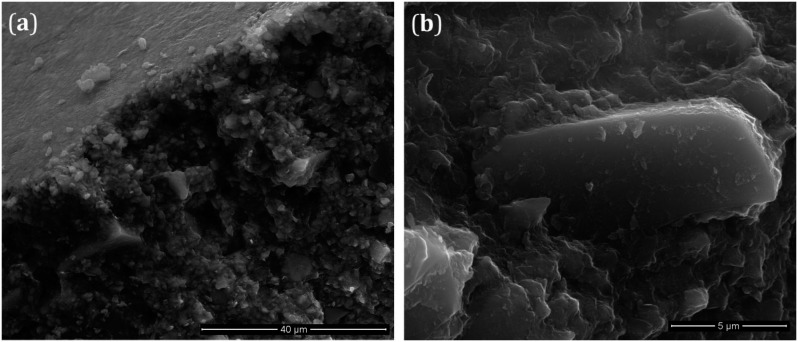
Scanning electron microscopy (SEM) micrographs of G-DU taken at 3,000× (a) and 15,000* (b) magnification

SEM micrographs of fractured G-DU as representative samples, acquired at 3000x and 15000x magnitudes, are shown in Figures 5a and 5b, respectively. As seen in Figure 5a, the top surface of the sample is quite smooth and there is no roughness and/or extra surface cracks. It can also be noticed that the glass filler particles are homogenously dispersed into the resin matrix. Based on the cracking crosssection, seen in Figure 5a, it can be assumed that the failure mechanism possibly followed the formation of a microcrack, then the rapidly growth of such weak domain under loading conditions over the breaking stress. However, it can also be inferred that the formation and growing of cracks occurred in the resin phase. A large and rectangular glass filler particle embedded into the resin phase is shown in Figure 5b. The average lateral size of glass particle is about 5-10 μm. An important observation in this micrograph is the excellent interfacial adhesion between the filler particle and the resin matrix.

### Surface hardness

**Figure 6:**
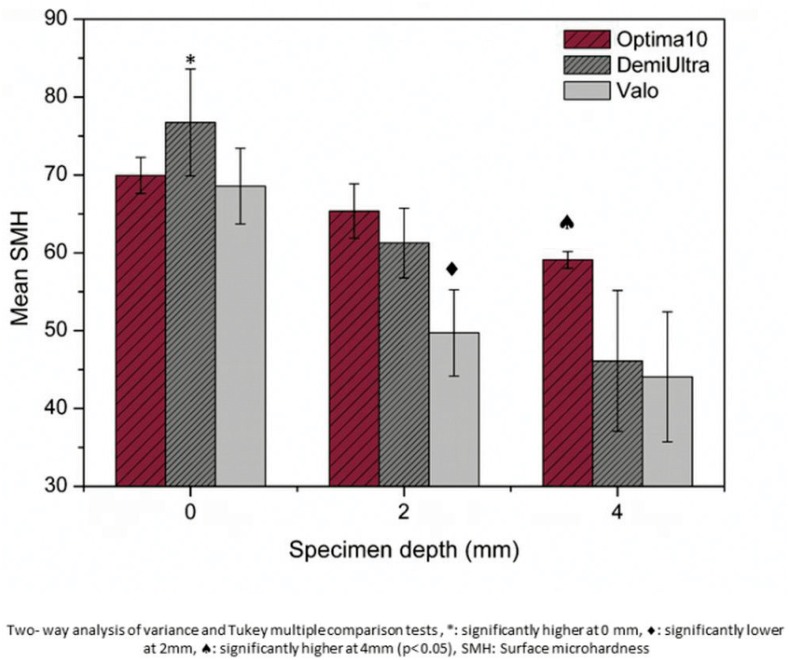
Surface microhardness values of samples

Average SMH values (±standard deviation) of samples were found to be 69.93 (±2.44), 76.74 (±7.29), and 68.56 (±5.13) at top surfaces (0 mm); 65.36 (±3.89), 61.26 (±4.98), and 49.72 (±6.20) at 2 mm depth; and 59.09 (±1.19), 46.13 (±10.11), and 44.06 (±9.32) at 4 mm depth for G-Opt, G-DU, and for G-Valo, respectively. The two-way ANOVA revealed that the light-curing device (F_2,51_=535.43, p<0.01), specimen depth (F_2,51_=2595.27, p<0.01) and interaction (F_4,51_=215.18, p<0.01) showed statistically significant effect on the SMH results (Figure 6). It was found that microhardness values of samples decreased with the increasing of specimen depth.

Relative change in SMH values were found to be 93.28% (G-Opt), 81.36% (G-DU), and 71.66% (G-Valo) for 2 mm, and 84.78% (G-Opt), 60.23% (G-DU), and 65.57% (GValo) for 4 mm.

### Mechanical properties of samples

**Figure 7:**
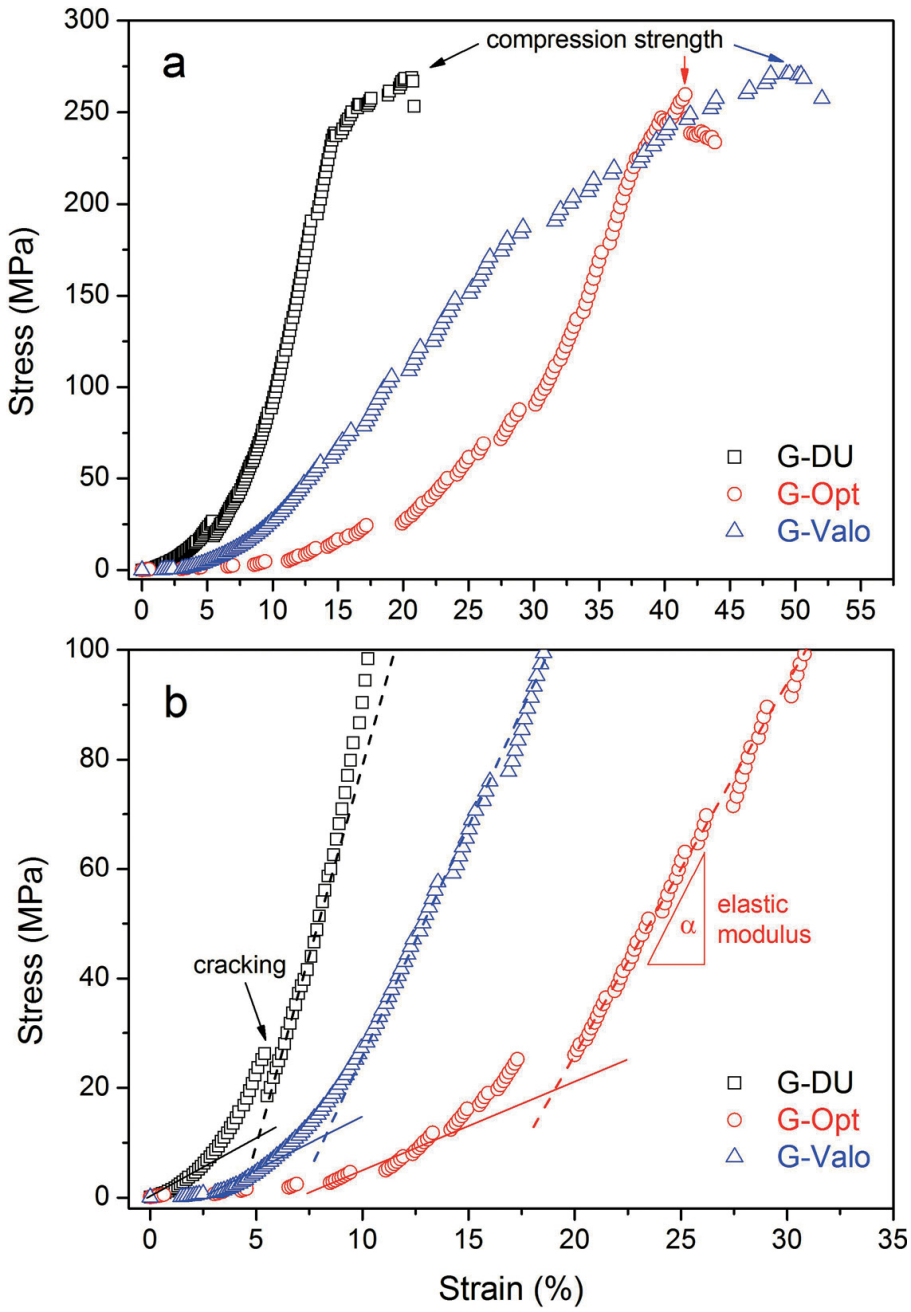
Representation of “stress-strain (SS) curves” of samples recorded during the compression test given in full SS range (a) and in initial elastic and transition region (b)

**Figure 8:**
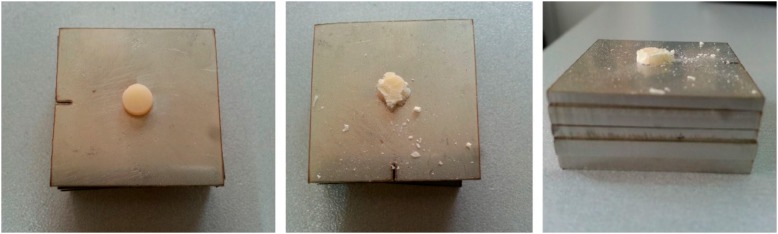
Compression test specimen before and after test

Typical stress-strain (SS) curves of samples recorded during the compression test are shown in Figure 7. The given SS curves represent the characteristic mechanical parameters of samples such as "elastic (or compressive) modulus", which is a slope of the SS curve, "compression strength", which is a maximum stress value just before the failure (or breaking), and "strain at break" under compressive loads (Table 2). Representative photographs of a test specimen before and after compression test are shown in Figure 8.

**Table 2:** Elastic modulus, compressive strength values of the samples

Sample code	Compression strength (MPa)	Elastic modulus (MPa)		Strain at break (%)
		**1^st^ region**	**2^nd^ region**	
G-DU	260 (±12.3)	818 (±110)	3533 (±168)	21.0 (±2.6)
G-Opt	274 (±18.4)	317 (±61)	1840 (±73)	39.8 (±3.4)
G-Valo	266 (±19.0)	255 (±62)	1635 (±171)	48.6 (±6.8)

Average FS and standard deviation values of samples are reported in Table 3. It was found that samples cured with the Optima 10 and the Demi Ultra exhibited similar FS values, while the sample cured with Valo had lower FS than the other devices.

**Table 3:** Comparison of flexural strength between giomer cured with different light-curing units (LCUs)

Sample	FS (MPa)	p
G-DU	111.54±6.94*	
G-Opt	118.86±8.26*	0.08
G-Valo	91.53±16.85	

One-way ANOVA used Tukey multiple comparison results.

* different from Valo (p<0.05)

## Discussion

Since their introduction into the dental market, LCUs have been regularly improved by manufacturers to provide better and faster polymerization. In this study, a giomer-based bulk fill dental restorative composite was polymerized with various curing protocols with different irradiance output, curing time and energy density. Structural and physical properties of samples reported previously indicate that different curing protocols affect chemical and solid-state properties of giomer-based composites. Thus, our null hypothesis was rejected and discussed below.

### Microstructure and morphology

The intensity of the characteristic absorption peak of unsaturated aliphatic C=C double bond originated from the methacrylate group at 1636 cm^−1^ and that of aromatic C=C double bond at 1604 cm^−1^ can be used to quantify the DC%, as previously described^21^. Another characteristic -COO-NH- stretching peak originated from UDMA is clearly seen at 1716 cm^1^. This peak is possibly superimposed with the characteristic peaks of ester (-COO-) and carbonyl (C=O) groups appearing in the wave number region of 1700-1730 cm^−1^. In this study, the DC% value was determined by considering the intensities of aliphatic and aromatic C=C bonds because while this number decreased due to the crosslinking reactions between the methacrylate groups of monomers, the aromatic C=C bonds originated from phenyl groups remained unchanged. In some studies, characteristic absorption peaks of aliphatic C=C and C=O bonds were used to calculate the DC% value^12^. However, it could be concluded that the peak intensity of ester or carbonyl is highly speculative for a quantitative determination, since these peaks can be influenced by the interfacial interaction or the adhesion between the resin phase and filler particles. Ilie and Fleming^15^ (2015) compared physical properties of a bulk-fill giomer and two resin- based bulk fill composites cured by a single LED device. They reported that giomer exhibited lower DC values than resin-based bulk fill composites due to its higher filler content.

In this study, SEM image of polymerized resin (Figure 5) implied that the resin phase successfully covered all filler surfaces and no holes or microvoids at the interfacial region appeared. This result also represents that the possible cracking mechanism mentioned before is originated from the cracking of the crosslinked resin phase rather than the particle or interfacial failure.

### Surface hardness

It is a well-known fact that when light is applied to a resin material, the irradiance decreases as it is reflected, dispersed and attenuated by the surface layers. Thus, deeper layers are generally less polymerized^7^. LCUs do not merely emit total energy to bulk structure of restorative material for photoinitiator activation. Uniform distribution of emitted energy in all layers of restorative material has been reported as crucially important to produce sufficient numbers of free radicals for adequate polymerization^22^.

According to previous studies, sufficient polymerization requires an array of different energy density levels. Some authors reported that it should be 16 J/cm^2^ or 21-24 J/cm^2^ for resin composites with 2 mm^4^ ^,^ ^7^. However, considering the compositional difference originated from different amounts of photoinitiators, fillers, monomers, and coloring agents, a uniform polymerization energy level generally cannot be supplied^27^.

In recent years, many brands have improved new LCUs with a high irradiance output and alternative wavelength LEDs that can activate different photoinitiators other than CQ. These improvements in irradiance outputs and polywave LEDs have been investigated in detail by researchers, because this integrated design of different diodes may emit a nonuniform light beam both from spectral and irradiance aspects^2^ ^,^ ^22^ ^,^ ^24^. Although beam homogeneity is not as critical as lasers or optical fibers in LCUs, reducing the polymerization time - which could compensate for a non-uniform beam - may further cut off the resin from scattered light^22^ ^,^ ^27^. Ilie and Stark^16^ (2014) measured the amount of energy reaching the bottom of three different bulk fill resin restoratives with 6 mm of thickness and reported very low levels.

In this study, SMH results of G-Valo were significantly lower than their counterparts in all depths. Although high irradiance may be interpreted as a shorter curing time from a total energy density concept, optimum cure requires sufficient polymerization time and irradiance output. Our results indicate that curing time might be a more effective factor on the microstructure formation of resin phases based on the DC% and SMH results. Gonulol, Ozer and Tunc^11^ (2016) also reported that SMH values of giomer and compomer-based restoratives cured with Valo (extra power mode) were lower than those cured with a second-generation LED-LCU. Our findings about the relationship between SMH and curing conditions were very consistent with their results.

Beolchi, et al.^3^ (2015) compared light irradiance of different LCUs, curing time and distances to reach an energy density level of 16 J/cm^2^. Their results showed that Valo in extra mode emitted an average irradiance of 1979.82 (±20.18) mW/cm^2^ and provided 16 J/cm^2^ at 4 mm away from the light tip. They also reported that curing time should be at least 8 s for efficiently polymerizing the composite restorative. Considering the absorbed, reflected and attenuated light by giomer in our samples, it can be concluded that applied curing time (6 s) with the Valo device was probably not enough to reach sufficient DC%.

Polymerization levels at top surfaces of the specimens cured with Valo and Optima did not vary much, but DemiUltra yielded higher DC% than Valo and Optima. This result was interesting because the light tip was in direct contact with the composite material. Regarding the irradiance outputs of devices, it can normally be expected that the polymerization level of samples would be similar since output values of these devices were well above the minimal 400 mW/ cm^2^ reported in the literature^2^ ^,^ ^22^. On the other hand, it is obvious that the wavelength of DemiUltra was closer to the characteristic absorbance of CQs (470 nm) than other devices. The higher DC% values of G-DU at top surface could be attributed to this.

The manufacturer of the restorative used in this study recommended a polymerization time of 10 s with a single peak Blue LED-LCU (440-490 nm, Light intensity: 1000 mW/cm^2^ or more). Although DU met the requirements of the recommended specifications and it was applied for 10 s, its SMH ratio was below 80% for the 4 mm sample. However, G-Opt samples were sufficiently polymerized, probably due to longer curing times.

Ilie and Stark^16^ (2014) determined relative change in SMH in three high viscosity bulk-fill composites in various curing conditions with Valo LCU. All three restoratives could maintain sufficient relative change in SMH with thicker increments than our samples. The authors attributed the rank of relative change in SMH to their refractive properties. It can also be generally accepted that this relative change in SMH is related to the polymerization efficiency and would indicate DC% depending on compositional variations or processing (curing) conditions. Although the filler volume and weight in the Ilie and Stark^16^ (2014) study is somewhat similar to bulk-fill giomer, the relative change in SMH difference between these bulk-fill restorative materials may be due to their respective translucency properties.

### Mechanical properties of samples

As seen in Figure 7b, all SS curves showed two distinctive regions. At the beginning of test (1^st^ region), matrix phase probable responses to compression loads onto sample disc. Shape or slope of curve in this region depends on the strength of matrix phase, which is directly related to the degree of crosslinking or curing (DC%). It can be expected that a sample having a higher DC% yields higher slope in SS curve, which corresponds to higher modulus in this region. It was found that G-DU sample showed much higher elastic modulus than other samples in 1^st^ region. This result is very consistent with the findings of DC% calculation and hardness test at 2 mm. Then, microcracks can emerge into sample with increasing of compression loads, as marked in Figure 7b. In the second region, SS curve goes with a much higher slope as compression loads were transferred to filler particles. It was found that G-DU sample showed a modulus (E) value about 3.5 GPa, while E values of other samples were lower than 2.0 GPa in this region. It was also proven that CS values of samples did not vary much depending on the curing device and protocol. This was possibly due to the fact that all samples included the same amount of filler and that the effect of curing protocol was more pronounced on DC% values rather than compression strength. On the other hand, it was found that strain at break values of samples cured with different light sources were significantly varied. Strain at break values of samples increased in the order of G-DU<G-Opt<G-Valo. This result was consistent with the relationship between DC% values and curing protocol. A lower DC% resulted in higher strain at break, as expected.

A standard flexural strength test method is commonly used for determining mechanical performance of dental restoratives and relates to fracture in clinic^10^. Although it has been suggested that samples having a thickness value below 4 mm do not represent clinical application condition of bulk- fill restoratives^9^, various studies have been reported on flexural test results of bulk-fill restorative samples with a size of 2×2×25 mm^14^ ^,^ ^20^. Abouelleil, et al.^1^ (2015) compared FS values of their test specimens to that of a standard ISO bulk-fill composite sample with a thickness of 4 mm, reporting that there was no significant change in FS values between 2 and 4 mm thick samples. It can be technically expected that the FS value of a specimen is normally independent from sample thickness if reported as unit of stress (MPa or GPa) rather than force (N, dyne or kgf).

According to the ISO 4049 standard, the FS values of dental restoratives classified as Type I (class of 1, 2, and 3) should be equal or greater than 80 MPa^17^. It was found that all the samples considered in our study met the FS requirements, which also implied that they could be safely used in load-bearing areas. Furthermore, the FS values measured in this study are also very consistent with the previously reported value (106.0±12.7 MPa) by Ilie^13^ (2016) for the same giomer-based composite polymerized with a LED-LCU for 20 s.

## Conclusion

In this study, microstructural features and mechanical properties of giomer-based bulk-fill restorative were quantified depending on polymerization efficiency of different curing devices and protocols. Curing device and applied protocol significantly affect DC%, surface hardness values and mechanical performances of composites under relatively low force conditions (load or stress) rather than high and destructive forces. Consequently, compressive strength of giomer bulk-fill restorative mainly depends on the amount of fillers and curing time is a more effective parameter than the power of device.

## References

[1] Abouelleil H, Pradelle N, Villat C, Attik N, Colon P, Grosgogeat B (2015). Comparison of mechanical properties of a new fiber reinforced composite and bulk filling composites. Restor Dent Endod..

[2] AlQahtani MQ, Michaud PL, Sullivan B, Labrie D, AlShaafi MM, Price RB (2015). Effect of high irradiance on depth of cure of a conventional and a bulk fill resin-based composite. Oper Dent..

[3] Beolchi RS, Moura-Netto C, Palo RM, Rocha Gomes Torres C, Pelissier B (2015). Changes in irradiance and energy density in relation to different curing distances. Braz Oral Res..

[4] Calabrese L, Fabiano F, Bonaccorsi LM, Fabiano V, Borsellino C (2015). Evaluation of the clinical impact of ISO 4049 in comparison with miniflexural test on mechanical performances of resin based composite. Int J Biomater..

[5] Capan BS, Akyuz S (2016). Current fluoride-releasing restorative materials used in pediatric dentistry. Clin Exp Health Sci..

[6] Chesterman J, Jowett A, Gallacher A, Nixon P (2017). Bulk-fill resin-based composite restorative materials: a review. Br Dent J..

[7] Della Bona A, Rosa V, Cecchetti D (2007). Influence of shade and irradiation time on the hardness of composite resins. Braz Dent J..

[8] Dionysopoulos D, Tolidis K, Gerasimou P (2016). The effect of composition, temperature and post-irradiation curing of bulk fill resin composites on polymerization efficiency. Mater Res..

[9] El-Damanhoury H, Platt J (2014). Polymerization shrinkage stress kinetics and related properties of bulk-fill resin composites. Oper Dent..

[10] Ferracane JL (2013). Resin-based composite performance: are there some things we can't predict?. Dent Mater..

[11] Gonulol N, Ozer S, Tunc ES (2016). Effect of a third-generation LED LCU on microhardness of tooth-colored restorative materials. Int J Paediatr Dent..

[12] Guerra R, Durán I, Ortiz P (1996). FTIR monomer conversion analysis of UDMA-based dental resins. J Oral Rehabil..

[13] Ilie N (2016). High viscosity bulk-fill giomer and ormocer-based resin composites: an *in-vitro* comparison of their mechanical behaviour. Stoma Edu J..

[14] Ilie N, Bucuta S, Draenert M (2013). Bulk-fill resin-based composites: an *in vitro* assessment of their mechanical performance. Oper Dent..

[15] Ilie N, Fleming GJ (2015). *In vitro* comparison of polymerisation kinetics and the micro-mechanical properties of low and high viscosity giomers and RBC materials. J Dent..

[16] Ilie N, Stark K (2014). Curing behaviour of high-viscosity bulk-fill composites. J Dent..

[17] Kara O, Atay A, Guven ME, Ismatullaev A, Usumez A (2016). Evaluation of curing distance of high intensity led curing units on microleakage of ceramic restorations [internet]. Selcuk Dental Journal..

[18] International Organization for Standardization (2009). ISO 4049:2009: Dentistry polymer-based filling, restorative and luting materials.

[19] Leprince J, Devaux J, Mullier T, Vreven J, Leloup G (2010). Pulpal-temperature rise and polymerization efficiency of LED curing lights. Oper Dent..

[20] Leprince JG, Palin WM, Vanacker J, Sabbagh J, Devaux J, Leloup G (2014). Physico-mechanical characteristics of commercially available bulk-fill composites. J Dent..

[21] Mendes LC, Tedesco AD, Miranda MS (2005). Determination of degree of conversion as function of depth of a photo-initiated dental restoration composite. Polym Test..

[22] Michaud PL, Price RB, Labrie D, Rueggeberg FA, Sullivan B (2014). Localised irradiance distribution found in dental light curing units. J Dent..

[23] Mobarak E, Elsayad I, Ibrahim M, El-Badrawy W (2009). Effect of LED lightcuring on the relative hardness of tooth-colored restorative materials. Oper Dent..

[24] Price RB, Labrie D, Rueggeberg FA, Felix CM (2010). Irradiance differences in the violet (405 nm) and blue (460 nm) spectral ranges among dental light-curing units. J Esthet Restor Dent..

[25] Rejman DJ, Eliades T, Bradley TG, Eliades G (2008). Polymerization efficiency of glass-ionomer and resin adhesives under molar bands. Angle Orthod..

[26] Rueggeberg FA (2011). State-of-the-art: dental photocuring - a review. Dent Mater..

[27] Shimokawa CA, Turbino ML, Harlow JE, Price HL, Price RB (2016). Light output from six battery operated dental curing lights. Mater Sci Eng C Mater Biol Appl..

